# A cavernous hemangioma in the small intestine of a teenage boy: a case report and literature review

**DOI:** 10.3389/fped.2025.1654913

**Published:** 2025-09-12

**Authors:** Tian Xie, Yi Long

**Affiliations:** Department of Children’s Medical Center, Hunan Provincial People’s Hospital, The First Affiliated Hospital of Hunan Normal University, Changsha, China

**Keywords:** cavernous hemangioma, hematochezia, children, anemia, multidisciplinary collaboration

## Abstract

**Background:**

A cavernous hemangioma in the small intestine is rare. Due to its hidden location and non-specific clinical symptoms, it is difficult to diagnose accurately. Here, we describe a 14-year-old boy with a cavernous hemangioma and bleeding at the junction of the jejunum and ileum.

**Case summary:**

The patient presented with clinical manifestations including melena, dizziness, fatigue, pale complexion, and shock. Abdominal contrast-enhanced computed tomography identified a hyperdense lesion within the small bowel lumen. Digital subtraction angiography (DSA) revealed aneurysmal changes in the mid-to-lower abdominal branches of the ileojejunal artery, which were suggestive of a hemorrhage originating from a small intestinal hemangioma. However, subsequent DSA-guided interventional embolization failed to achieve hemostasis. Following a hospital-wide multidisciplinary consultation, a laparoscopic exploration was conducted. During the procedure, a 1.5 cm × 1.5 cm mass was detected at the ileojejunal junction and successfully resected. The postoperative pathological examination confirmed the lesion to be a cavernous hemangioma. One week after surgery, the patient's hemoglobin level increased to 86 g/L, with no recurrence of bloody stools. At the 1-month follow-up, no signs of disease recurrence were observed.

**Conclusion:**

This case report presents significant clinical implications for the diagnosis and management of small intestinal cavernous hemangiomas in the pediatric population.

## Introduction

A small intestinal hemangioma is a rare vascular malformation, accounting for 7%–10% of all benign small intestinal tumors. Approximately 60% of cases arise in the jejunum, whereas involvement of the ileum is less frequent (1, 2). Histopathologically, these lesions are congenital vascular malformations and are categorized into capillary, cavernous, or mixed subtypes, with the cavernous variant being the most prevalent (3).

Clinically, patients may present with symptoms such as abdominal pain, gastrointestinal bleeding, intestinal obstruction, intussusception, and intestinal perforation (3–6). Owing to their non-specific clinical manifestations and the absence of definitive ancillary test findings, a misdiagnosis or missed diagnosis is common. Although recent advancements in small bowel endoscopy have enhanced detection rates, the limited accessibility to this technology across hospitals remains a contributing factor to diagnostic delays—especially in the case of rare small intestinal cavernous hemangiomas.

## Case description

A 14-year-old male patient presented with a 10-day history of melena (small in amount), accompanied by dizziness, fatigue, pallor, and low-grade fever (maximum temperature of 37.7°C). He denied a history of chills, abdominal pain, or perianal pain.

Prior to admission, he underwent a gastroscopy at another hospital, which revealed chronic non-atrophic gastritis with bile reflux and erosions; concurrent laboratory testing showed a hemoglobin level of 51 g/L. The initial diagnosis at the external hospital was “iron deficiency anemia or hemorrhagic anemia,” but treatment with a blood transfusion and iron supplementation failed to improve his symptoms or anemia indices.

One day before admission, the patient developed bright red hematochezia and was subsequently transferred to our hospital. He had an unremarkable medical history and no significant medication use.

Upon admission, the patient underwent a comprehensive workup, including coagulation function tests, anemia-related panels, liver and kidney function tests, cardiac enzymes, and electrolyte assessments; all the results were within the normal reference ranges.

Despite active interventions, including fasting, administration of hemostatic agents, blood transfusion, and supportive care to stabilize vital signs, the patient continued to have persistent melena, with a progressive downward trend in hemoglobin levels.

**Figure 1:**
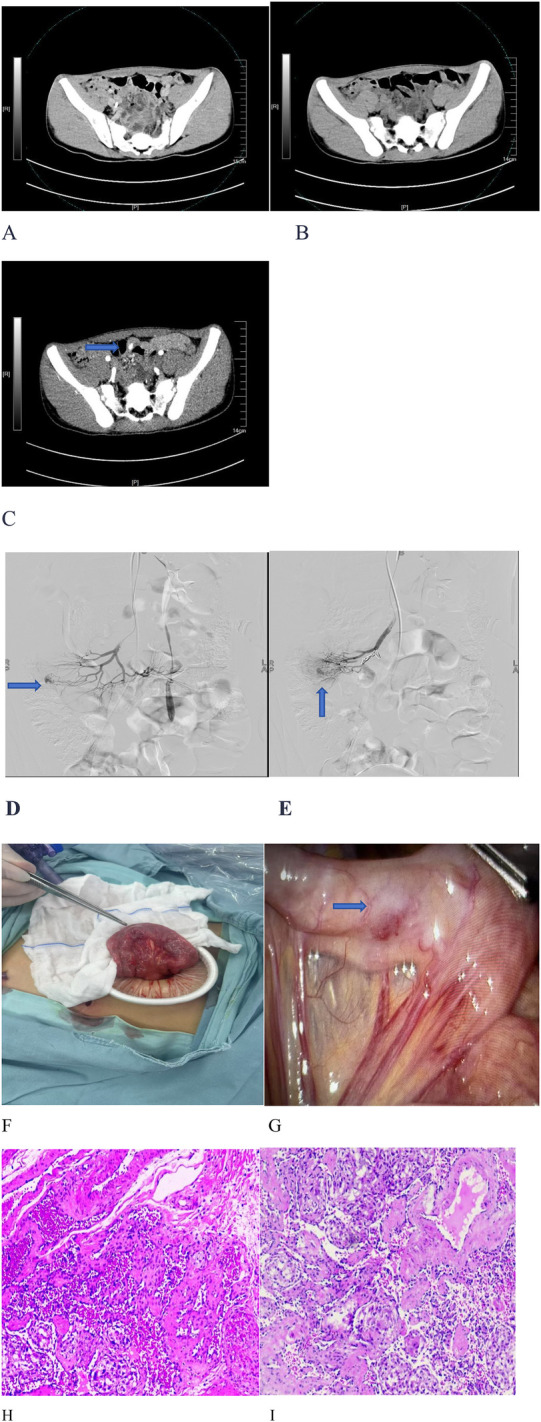
Imaging, angiography, surgery, and pathology of an ileal cavernous hemangioma. **(A)** No obvious lesion was identified on unenhanced phase imaging. **(B)** No abnormalities were observed in the portal venous phase. **(C)** A hyperattenuating lesion was detected during the arterial phase. **(D,E)** Superior mesenteric artery angiography revealed contrast extravasation from an ileojejunal artery branch, consistent with an active small bowel hemorrhage. **(F,G)** Laparoscopic exploration revealed a 1.5 cm × 1.5 cm mesenteric-based mass at the junction of the jejunum and ileum, which had a smooth and erythematous surface. **(H,I)** Hematoxylin-eosin staining demonstrated dilated congested sinusoidal spaces within the submucosa.

Abdominal contrast-enhanced CT revealed a hyperattenuating lesion in the small bowel of undetermined etiology (Figure 1C). Both a gastroscopy and a colonoscopy showed no abnormalities, indicating a small intestinal bleeding source. Scintigraphy for ectopic gastric mucosa was negative.

The Interventional Radiology Department recommended digital subtraction angiography (DSA) of the superior mesenteric artery. This examination showed contrast extravasation from the ileojejunal artery branches (Figures 1D,E), confirming an active small intestinal hemorrhage. A small intestinal hemangioma was initially suspected; however, after coil embolization of the ileojejunal artery branches, definitive hemostasis was not achieved.

Due to the child’s significant hemorrhage, the Pediatric Surgery Service recommended diagnostic and therapeutic laparoscopic exploration. Intraoperatively, a 1.5 cm × 1.5 cm mesenteric-based lesion with a smooth, erythematous surface was identified at the junction of jejunum and ileum (Figures 1F,G). The bleeding lesion was completely resected (Figures 1F,G). The final pathological examination confirmed it was a cavernous hemangioma (Figures 1H,I).

## Physical examination

Upon admission, the patient’s heart rate was 128 beats/min with a blood pressure of 91/52 mmHg (converted from 12.1/6.9 kPa). Pallor was noted, and all other physical examination findings were unremarkable.

## Laboratory examinations

Laboratory tests revealed severe normocytic anemia (hemoglobin: 5.0 g/dL) with a positive fecal occult blood test. No abnormalities were detected in liver and kidney function, coagulation profiles, or bone marrow aspiration smears.

## Imaging examinations

Computed tomography (SOMATOM Definition Flash, Siemens, Germany) revealed an arterial-phase enhancing lesion in the small intestine (Figures 1A–C). No abnormalities were found in gastroscopy and colonoscopy. DSA (Figures 1D,E) was conducted under ultrasound guidance, with the modified Seldinger technique used to puncture the right femoral artery and insert a catheter sheath.

• Abdominal aortography revealed thickening and tortuosity of the intrahepatic arteries, with multiple clustered nodules throughout the liver suggestive of hemangiomas. No active extravasation was observed.
• Superior mesenteric angiography demonstrated patent main trunks but disorganized terminal branches without active bleeding.
• Microcatheters were sequentially superselected into jejunal and ileal artery branches, and angiography showed no bleeding.

After positioning the patient in a 30° right anterior oblique position (aligned with the prior CT scan for anatomical correlation), repeat angiography identified contrast extravasation from a jejunoileal artery branch in the mid-lower abdomen. Following superselection with a microguidewire-confirmed catheter, the branch was found to have aneurysmal changes with an active hemorrhage.

Coil embolization was performed; however, the therapeutic effect was suboptimal.

## Treatment

Laparoscopic exploration was performed to expose the ileocecal region. The exploration progressed sequentially from the ileocecal region. At the ileojejunal junction, a 1.5 cm × 1.5 cm mass with a smooth, erythematous surface was identified. Its base was attached to the mesentery. The mesenteric plane surrounding the mass was dissected toward its base using a harmonic scalpel. Multiple mesenteric vessels were visualized at the base. Consequently, conversion to laparotomy was performed for small bowel resection of the mass, followed by intestinal anastomosis.

A microscopic examination revealed twisted and dilated thin-walled vascular channels within the submucosa at the hemorrhagic site. The definitive pathological diagnosis was a cavernous hemangioma at the ileojejunal junction.

## Outcome and follow-up

The patient recovered quickly postoperatively with no further bleeding. At the 1-month follow-up, his hemoglobin levels had increased to 8.6 g/dL (normal range: 12–16 g/dL).

## Discussion

In 1839, Philips first described gastrointestinal bleeding caused by vascular tumors. Since the 1950s, with the development of diagnostic techniques such as angiography, the number of reported cases of small intestinal hemangiomas has increased (7). The etiology of this disease is unknown, and it affects people of all age groups [no gender difference, with a high incidence in adolescents and adults around 30 years old (8)]. The lesions are usually located in the jejunum and ileum (can be single, multiple, or diffusely involved). The main symptoms are recurrent gastrointestinal bleeding and iron-deficiency anemia (accompanied by dizziness, fatigue, etc.). Early diagnosis is difficult due to atypical manifestations; complications include intussusception (9), and a definitive diagnosis relies on a pathological examination. For lower gastrointestinal bleeding in children, juvenile polyps and other conditions should be ruled out first, with colonoscopy the preferred examination.

### Case examination and diagnosis

In this case, CT showed contrast extravasation in the arterial phase (suggesting active small intestinal bleeding), but no abnormalities were found in the venous and portal phases. Moreover, CT has low specificity in diagnosing vascular lesions (1). Common examinations for small intestinal bleeding include DSA and capsule endoscopy (CE) (10, 11). Since enteroscopy and CE were not available in our hospital, DSA was performed [the localization accuracy for bleeding ≥0.5 mL/min is 50%–72%, with simultaneous hemostasis possible; the diagnostic rate decreases to 25%–50% in a case with slow bleeding (12)]. DSA detected bleeding from the jejunoileal artery branch, and embolization failed. Different from the traditional diagnostic method relying on enteroscopy (1), DSA provides a new option for bleeding localization.

Endoscopic resection or embolization may be an option in some cases (13), but this pediatric patient required surgery due to acute massive bleeding leading to severe anemia and failed interventional therapy. CE is non-invasive but cannot be used for biopsy or localization (14, 15), while double-balloon enteroscopy (DBE) allows for biopsy and treatment but is invasive (16). Based on the CT, DSA (suspected vascular malformation), and high bleeding rate, laparoscopic exploration was selected in this case. A 1.5 cm × 1.5 cm mass was resected from the mid-jejunum, and postoperative pathology confirmed a small intestinal cavernous hemangioma (Fuhrman grade I–II).

The diagnosis of small intestinal cavernous hemangiomas is challenging because the lesions are usually located in the mid-jejunum and intestinal peristalsis interferes with localization. Selective mesenteric angiography is the first choice (it can detect bleeding ≥0.5 mL/min and simultaneous embolization can be performed, with the cessation of contrast extravasation the criterion for a successful embolization). If embolization fails (failure rate: 15%–30%), surgery is recommended; lesions >2 cm are easily identifiable during surgery. Segmental intestinal resection yields good results, with a 5-year recurrence rate of <5%.

## Data Availability

The original contributions presented in the study are included in the article/Supplementary Material, further inquiries can be directed to the corresponding author.
